# Impact factor to discovery: The future of thrombosis and hemostasis research

**DOI:** 10.1002/rth2.12791

**Published:** 2022-08-26

**Authors:** Mary Cushman

**Affiliations:** ^1^ Larner College of Medicine University of Vermont Burlington Vermont USA

**Keywords:** COVID‐19, hemostasis, impact factor, journal citation indicator, thrombosis

The COVID‐19 pandemic saw a storm of new science, especially in our thrombosis and hemostasis community. Were you involved in this? What will you do next? In this editorial, I report on the first journal impact factor for *Research and Practice in Thrombosis and Haemostasis* (*RPTH*), a new citation metric we are watching, and how our research community can move forward to future important discoveries.

**FIGURE 1:**
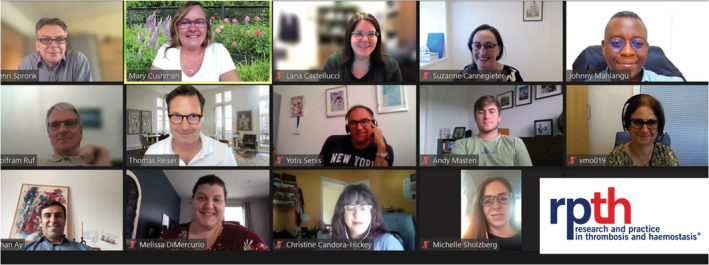
*Research and Practice in Thrombosis and Haemostasis* impact factor watch party, 2022

As editor in chief of *RPTH*, my team and I were thrilled that the journal received its first impact factor in June 2022 on the occasion of our fifth birthday (Figure 1). We were even more thrilled that the impact factor of 5.953 placed us in excellent position among journals in our field; we rank 16 of 67 journals in the peripheral vascular disease category and 24 of 79 journals in the hematology category. These placings surpassed my imaginings when the journal launched in 2017. Then, I simply wanted to publish the best science and hope that we would receive an impact factor in a reasonable time frame.

Table 1 shows journal impact factors from last year and this year for journals from the two categories above that emphasize thrombosis and hemostasis topics. It is notable that nearly all the impact factors rose, some substantially. Our “sister journal,” *Journal of Thrombosis and Haemostasis*, experienced the largest increase by far, and it topped the list this year. *RPTH* ranked 6 of 14 of these journals for this year's impact factor.

**TABLE 1:** Journal impact factors for 2020 and 2021 for journals emphasizing thrombosis and hemostasis

Journal Name, in order of 2021 impact factor	2020 Impact Factor	2021 Impact Factor	Fold‐change
*Journal of Thrombosis and Haemostasis*	5.824	16.036	+2.75
*Arteriosclerosis Thrombosis and Vascular Biology*	8.311	10.514	+1.26
*Thrombosis Research*	3.944	10.407	+2.64
*Thrombosis and Hemostasis*	5.249	6.681	+1.27
*Seminars In Thrombosis and Hemostasis*	4.180	6.398	+1.53
*Research and Practice in Thrombosis and Haemostasis*	n/a	5.953	n/a
*Thrombosis Journal*	5.500	5.509	1.00
*Journal of Thrombosis and Thrombolysis*	2.300	5.221	+2.27
*Vascular Medicine*	3.530	4.739	+1.34
*Journal of Atherosclerosis and Thrombosis*	3.700	4.394	+1.19
*Haemophilia*	4.287	4.263	−1.01
*Platelets*	3.862	4.236	+1.10
*Clinical and Applied Thrombosis‐Hemostasis*	2.389	3.512	+1.47
*Blood Coagulation & Fibrinolysis*	1.276	1.061	−0.83

Impact factor is a metric often used by authors to help them decide if a journal might be a good home for their research, and, unfortunately, despite its weaknesses, many universities use it to evaluate faculty for promotion.

**FIGURE 2:**
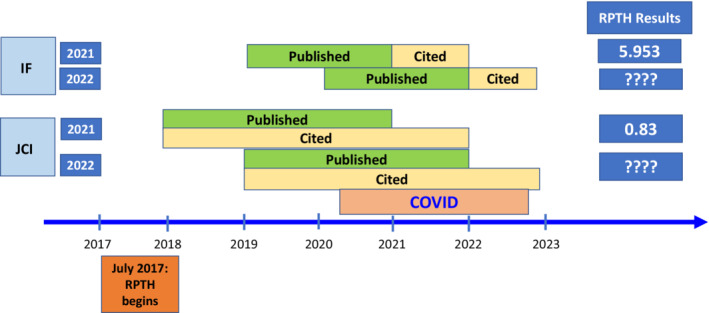
Calculation of the Journal Impact Factor (IF) and the Journal Citation Indicator (JCI)

As a refresher, impact factor is calculated annually by Clarivate Analytics. As shown in Figure 2, its value for 2022 is based on all articles published in calendar years 2019 and 2020, and the number of citations those articles receive in calendar year 2021. This number of citations is divided by the number of “citable items” published in the 2‐year period. Citable items generally include research articles, proceedings, and review articles. Items not counted in the denominator include editorial works, commentaries, and letters. As such, some highly cited works may not be counted in the denominator.

There are other metrics that quantify journal impact. The “journal citation indicator” (JCI) metric was released by Clarivate Analytics for the first time in 2021.^1^ Unlike impact factor, this metric is given to all journals in the Web of Science Core Collection; a journal does not have to be accepted by Clarivate first, as is the case for impact factor. The JCI field‐normalizes citation rate of a journal to the field of study of that journal and the document type. In this regard, it might be more useful to authors considering which journal to submit to, and moreover, more useful to assessment of faculty progress by their institutions as it considers the field of study the person is involved with. The JCI is the mean of the Category Normalized Citation Impact of each article that is published. The Category Normalized Citation Impact considers both the field of study and the article type (review articles are indexed to review articles for example). A JCI value of 1.0 means that articles in a journal received a number of citations equal to the average citation count of articles in journals in that subject category. Shown in Figure 2, the timeline for the JCI calculation differs from the timeline for the impact factor. Articles and reviews published in the previous 3 years are considered in the JCI; for example, the 2021 metric just released in 2022 includes articles published in 2018, 2019, and 2020, and counts citations to those articles starting from the time of publication through the calendar year 2021. As a result, the JCI can be considered a more stable metric than the impact factor, and it accounts for early and later citations.

In 2021, *RPTH* received a JCI of 0.75, ranking 31 of 93 hematology journals (including a number of journals without an impact factor), and first among several hematology journals without an impact factor. We were at a “disadvantage” for this JCI because we only had a few issues in our inaugural year of 2017, which contributed to that year's JCI. In 2022, the JCI improved to 0.83, ranking 30 of 95 journals in the hematology category. It remains to be seen whether the JCI will have staying power. A PubMed search for the term yielded no articles about it over the year since it was released.

One cannot write about this year's impact factor without considering the inflationary impact of COVID‐19, which we have seen in our field owing to the coagulopathy of COVID‐19. According to PubMed, more than 270,000 publications to date refer to COVID‐19 or SARS‐CoV‐2 published in 2020 or later. Accordingly, citations to these articles are growing rapidly. Many of these articles were published in 2020 and so will continue to contribute to impact factors released in 2023, along with all articles published in 2021. One might expect that all journals with impact factors that increased because of the surge of papers related to COVID‐19 will experience a decline in their impact factor after 2024, with the JCI lagging behind as it considers a 3‐year time window and compares across journals within our field.

What can we do to build on the momentum of discovery reflected in these changing journal citation metrics?

I call on our thrombosis and hemostasis research community to consider the vast new knowledge gained in our field during the pandemic, and how our community might leverage this research to build and sustain our field. Three ideas come to mind. First is expanded research on topics such as repurposing anticoagulant and other antithrombotic medications for nonthrombosis indications (including other infectious diseases perhaps), improved methods to identify medical patients at risk of thrombosis, and expanded basic and translational research on the roles of hemostatic and thrombotic pathways in organ failure syndromes (especially lung, heart, liver, and kidney), brain health, and aging phenotypes. Second, it is important to consider that scientific innovation during the pandemic has attracted new early career researchers to our field. Early‐career researchers often bring the most novel ideas for the future, and as an editor in chief promoting early careerists in research, I cannot wait to see the impact in this regard. Third, interest in coagulopathies has increased among nonhematologists involved in COVID‐19 care and research. Finding a home for these individuals in our field is important. This can be done through journal initiatives and Congress programming. *RPTH* is committed to publishing interdisciplinary research and engaging with individuals from outside hematology, for example.

The terrible events of the pandemic lead us to consider the new knowledge gained as a silver lining,^2^ and to be as innovative as possible in applying this new knowledge to the future. If you are new to the field of thrombosis and hemostasis research, I would love to hear from you about your needs.
